# Development and evaluation of a formula for predicting introduction of medication self-management in stroke patients in the Kaifukuki rehabilitation ward

**DOI:** 10.1186/s40780-016-0070-7

**Published:** 2017-01-10

**Authors:** Hisato Fujihara, Mari Kogo, Isao Saito, Nobuyuki Kawate, Masazumi Mizuma, Hiroko Suzuki, Jun-ichiro Murayama, Tadanori Sasaki

**Affiliations:** 1 Department of Pharmacy, Showa University Fujigaoka Rehabilitation Hospital, 2-1-1 Fujigaoka, Aoba-ku, Yokohama, Kanagawa 227-8518 Japan; 2 Department of Hospital Pharmaceutics, School of Pharmacy, Showa University, 1-5-8 Hatanodai, Shinagawa-ku, Tokyo, 142-8555 Japan; 3 Department of Pharmacy, Showa University Fujigaoka Hospital, 1-30 Fujigaoka, Aoba-ku, Yokohama, Kanagawa, 227-8501 Japan; 4 Department of Rehabilitation Medicine, School of Medicine, Showa University, 2-1-1 Fujigaoka, Aoba-ku, Yokohama, Kanagawa, 227-8501 Japan; 5 Department of Pharmacy, Showa University Hospital, 1-5-8 Hatanodai, Shinagawa-ku, Tokyo, 142-8666 Japan

**Keywords:** Stroke patient, Rehabilitation, Multivariate analysis, Predictive factor, Medication self-management, FIM

## Abstract

**Background:**

Medication self-management in stroke patients is important to prevent further progression of disease and incidence of side effects. The purpose of this study was to create a formula for predicting medication self-management introduction in stroke patients using functional independence measure items and patient data, including medication-related information.

**Methods:**

This was a retrospective analysis of 104 patients (cerebral infarction, cerebral hemorrhage, subarachnoid hemorrhage) discharged from the Kaifukuki rehabilitation ward at Showa University Fujigaoka Rehabilitation Hospital from January to December 2012. Multivariate analysis was performed to develop a formula for predicting achievement of medication self-management.

**Results:**

Of the 104 patients, 39 (37.5%) achieved medication self-management. In the logistic regression analysis, number of drugs, age, walk/wheelchair mobility FIM, and memory FIM were extracted as significant factors independently contributing to achievement of medication self-management (*p* < 0.05). The prediction formula was [4.404 − 0.229 × number of drugs at admission + 0.470 × walk/wheelchair mobility FIM at admission + 0.416 × memory FIM at admission − 0.112 × age].

**Conclusions:**

In the future, this formula may be used as an index to predict success of medication self-management in stroke patients.

## Background

Stroke patients admitted to a Kaifukuki rehabilitation ward often develop cognitive impairment. Therefore, there is a pressing need to improve patients’ abilities to self-manage their social life and activities. Previous reports have shown that establishing goals during the early phase of hospitalization hastens effective rehabilitation [1–3].

However, the medication self-management has not been appropriately evaluated for stroke patients, although it is important to prevent further disease progression and incidence of side effects.

Pharmacists who work in a Kaifukuki rehabilitation ward must support introduction of safe medication self-management and prevent medication errors in stroke patients. In addition, the mission of hospital pharmacists is to help patients achieve optimal medication self-management during hospitalization. However, decisions regarding whether to introduce medication self-management for stroke patients should be based on optimal objective indicators.

There are many studies reporting timing of discharge for stroke patients using admission data [4–15]. These reports use the functional independence measure (FIM), which objectively quantifies activities of daily living and is widely used in the rehabilitation ward as an evaluation criterion. Moreover, many studies used FIM items at admission to predict achievement of medication self-management in stroke patients [16–18]. However, there is currently no objective index for determining the likelihood of achieving medication self-management, including those measuring medication-taking behavior, such as number of drugs or number of doses per day.

The purpose of this study was to create a formula to predict if medication self-management would be effective for stroke patients using FIM items and patient data, including medication-related information.

## Methods

### Patients

The subjects included 104 patients (cerebral infarction, cerebral hemorrhage, subarachnoid hemorrhage) discharged from the Kaifukuki rehabilitation ward in Showa University Fujigaoka Rehabilitation Hospital from January to December 2012. A retrospective cohort study was conducted using data from the medical charts of the subjects. Subjects were excluded if they had a medication error during hospitalization after achievement of medication self-management. This study was approved by the ethics committee of Showa University Fujigaoka Hospital (approval no. 2012105).

### Clinical parameters

We collected data from the medical charts, including age, sex, post-onset rehabilitation hospital day, type of disease (cerebral infarction, cerebral hemorrhage, subarachnoid hemorrhage), number of drugs, number of doses per day, number of doses to be taken once only, one-dose packages, and FIM item score as scored by nurses at the inpatient ward.

### Standards to introduce medication self-management

Patients need to achieve all eight items shown in Table 1, and medical staff (physicians, pharmacists, nurses and occupational therapists) discuss and judge if safe medication self-management is applicable.

**Table 1:** Eight items that are necessary to introduce drug action

□ Do you know purpose of drugs?	YES/NO
□ Can you count the number of drugs?	YES/NO
□ Do you know when to take drugs?	YES/NO
□ Can you remember when to took drugs?	YES/NO
□ Can you bring drugs to the mouth?	YES/NO
□ Can you swallow drugs?	YES/NO
□ Could you management daily medication by yourself?	YES/NO
□ Could you continue taking drugs?	YES/NO

### Endpoint

The endpoint of this survey was achievement of medication self-management at discharge.

### Univariate analysis

We compared each variable between two groups: those achieving self-management and those who did not.

### Comparison of changes in the number of drugs and number of doses at admission, introduction of medication self-management, and discharge

To exclude the influence of changes in medicine in the hospital, we compared the number of drugs and number of doses between admission and discharge. In addition, we compared the number of drugs and number of doses per day between admission and at the start of medication self-management.

### Multivariate analysis and creation of a prediction formula

Parameters that were significantly different in the univariate analysis were entered in the multivariate analysis. Significant independent variables contributing to medication self-management were extracted using stepwise selection methods. In addition, we composed a formula to predict medication self-management by using extracted items along with the regression coefficient. The prediction formula was y = aX_1_ + bX_2_ + cX_3_, where y is the objective variable; X_1_, *X*_2_, and X_3_ are the explanatory variables; and a, b, and c are regression coefficients. We used backward stepwise multiple regression analysis to select the variables.

### Evaluation of the validity of the prediction formula

We evaluated the validity of the formula by using the degrees of freedom adjusted R^2^ statistic, lack of fit (LOF), and area under the receiver operating characteristic (ROC) curve, which provides an index indicating the association of the sensitivity and the specificity.

### Statistical analysis

To examine between-group differences, the *t*-test was used for continuous variables, Fisher’s exact test was used for categorical variables, and the Wilcoxon rank sum test was used for the FIM item score. A value of *p* < 0.05 was considered statistically significant. JMP^®^ v. 9.0 (SAS Institute Inc., Cary, NC, USA) was used for the statistical analyses.

## Results

### Patients characteristics

Table 2 shows the characteristics of all patients. The average age was 70.0 ± 12.3 years; 65 (62.5%) were men, and 39 (37.5%) were women. Of the 104 patients, 39 (37.5%) achieved medication self-management, and 65 (62.5%) patients did not.

**Table 2:** Characteristics of the patients : host-related factors (*n* = 104)

Variable	*n* (%) or Mean ± SD
Age (years)	70.0 ± 12.3
Sex	
Male	65 (62.5)
Female	39 (37.5)
Post-onset rehabilitation hospital day (days)	23.5 ± 9.4
Diagnosis	
Cerebral infarction	65 (62.5)
Cerebral hemorrhage	34 (32.7)
Subarachnoid hemorrhage	5 (4.8)

### Univariate analysis

Table 3 lists the results of the univariate analysis. Age, post-onset rehabilitation hospital day, number of drugs, and number of doses per day were statistically different between the self-management group and the non-self-management group (*p* < 0.05). All FIM items score were significantly different between the two groups (Table 3, *p* < 0.05).

**Table 3:** Comparison of variables between medication self-management and medication non-self-management groups

		SM (*n* = 39)	Non-SM (*n* = 65)	*P* value
		*n*, Mean ± SD	*n*, Mean ± SD	
Characteristics of the patients	Age (years)	62.2 ± 11.6	74.7 ± 10.1	<0.001
	Sex			0.536
	Male	26	39	
	Female	13	26	
	Post-onset rehabilitation hospital day (days)	23.5 ± 9.4	29.7 ± 20.5	0.040
Medication-related item	number of drug	4.4 ± 2.3	6.5 ± 3.3	<0.001
	number of doses per day	2.3 ± 1.4	3.0 ± 1.2	0.008
	number of dose of medicine to be taken only once	0.5 ± 1.1	0.7 ± 0.8	0.759
	one-dose packages/not one-dose packages	33/6	61/4	0.170
FIM item	Eating	6.2 ± 1.3	4.8 ± 2.0	<0.001
	Grooming	5.7 ± 1.3	3.8 ± 2.0	<0.001
	Bathing	4.7 ± 1.7	2.9 ± 1.9	<0.001
	Dressing upper body	4.9 ± 1.6	3.2 ± 1.9	<0.001
	Dressing under body	4.8 ± 1.7	2.9 ± 1.9	<0.001
	Toileting	5.6 ± 1.7	3.3 ± 2.3	<0.001
	Bladder	6.0 ± 1.8	4.0 ± 2.6	<0.001
	Bowel	5.7 ± 2.1	4.0 ± 2.5	<0.001
	Bed chair transfer	5.7 ± 1.4	3.7 ± 1.7	<0.001
	Toilet transfer	5.6 ± 1.5	3.5 ± 1.8	<0.001
	Tub shower transfer	4.7 ± 1.5	3.1 ± 1.7	<0.001
	Walk/wheelchair mobility	5.3 ± 1.9	2.7 ± 1.9	<0.001
	Stairs	2.3 ± 2.3	1.2 ± 0.9	0.002
	Comprehension	6.1 ± 1.2	4.6 ± 2.0	<0.001
	Expression	6.0 ± 1.5	4.8 ± 2.1	0.003
	Social interaction	6.7 ± 1.0	5.2 ± 2.2	<0.001
	Problem solving	5.7 ± 1.4	3.5 ± 2.1	<0.001
	Memory	5.9 ± 1.4	3.8 ± 2.0	<0.001

*SM* medication self-management group

### Comparison of changes in the number of drugs and number of doses from admission to discharge

There was no significant difference between admission and discharge in the number of drugs and number of doses. In the medication self-management group, there was no significant difference between admission and the introduction of medication self-management (Table 4).

**Table 4:** Comparison of changes in the number of drugs and number of doses from admission to discharge

		Admission	Self-management introduced taking the drug	*P value*	Discharge	*P value*
		Mean ± SD	Mean ± SD		Mean ± SD	
SM (*n* = 39)	Number of drugs	4.4 ± 2.3	4.8 ± 2.4	0.147	5.0 ± 2.6	0.068
	Number of doses per day	2.3 ± 1.4	2.3 ± 1.2	0.744	2.2 ± 1.1	0.680
non-SM (*n* = 65)	Number of drugs	6.4 ± 3.3	-	-	6.9 ± 2.7	0.084
	Number of doses per day	3.0 ± 1.2	-	-	3.1 ± 1.2	0.494

*SM* medication self-management group

### Multivariate analysis and formation of a prediction formula

In the logistic regression analysis, number of drugs, age, walk/wheelchair mobility FIM, and memory FIM were extracted as significant factors independently contributing to achievement of medication self-management in stroke patients (*p* < 0.05). Table 5 lists odds ratios and 95% confidence intervals. We created the prediction formula by extracting four factors and using the regression coefficient.

**Table 5:** Results of stepwise multiple regression analyses

Factor	Regression coefficient	Odds ratio	95% confidence interval	*P* value
Number of drugs	−0.229	0.795	0.629–0.969	0.035
Walk/wheelchair mobility FIM	0.470	1.600	1.186–2.251	0.004
Memory FIM	0.416	1.517	1.073–2.225	0.023
Age	−0.112	0.894	0.837–0.944	0.0002
Intercept	4.404			0.045

The formula was

$$ \begin{array}{l}\Big[4.404-0.229\times \mathrm{number}\;\mathrm{of}\;\mathrm{drugs}+0.470\times \mathrm{walk}/\mathrm{wheelchair}\;\mathrm{mobility}\ \mathrm{F}\mathrm{I}\mathrm{M}+0.416\\ {}\times \mathrm{memory}\;\mathrm{F}\mathrm{I}\mathrm{M}-0.112\times \mathrm{age}\Big].\end{array} $$

### Evaluation of the validity of prediction formula

In testing the validity of the prediction formula, the R^2^ value was 0.49, and the *P*-value of LOF was 0.987. The area under the ROC curve was 0.926. Thus, our prediction model showed high accuracy.

## Discussion

We created a formula to predict the likelihood of achievement of medication self-management for stroke patients using patient data, including items from the FIM item as well as drug-related information. It is often emphasized that pharmacists working in the Kaifukuki rehabilitation ward have an important role to support stroke patients to achieve medication self-management during hospitalization. In the current study, we confirmed the internal validity of a new prediction formula that may function as an appropriate index to predict whether patients will achieve medication self-management at discharge. Pharmacists will be able to use this formula to help provide appropriate instruction to stroke patients.

The multivariate analysis revealed that the number of drugs at admission greatly influenced medication self-management. There are some studies including each FIM item, but there is no report including drug-related information [16–18]. Accordingly, when pharmacists introduce medication self-management for stroke patients, it is important to consider the number of drugs at admission.

Moreover, to exclude the influence of changes in drugs occurring during hospitalization, we compared changes in the number of drugs and in the number of doses from admission to discharge. However, there were no differences. Previously, Sato et al. reported that the number of drugs during hospitalization decreased by 0.47 per patient with pharmacist intervention; thus, it may be difficult to further decrease the number of drugs during hospitalization in the Kaifukuki rehabilitation ward [19]. Regardless, based on the current data, it is possible to predict medication self-management using drug-related information data at admission.

This study suggests that the fewer drugs at admission, the more likely a patient is to achieve medication self-management. Some studies report the relation between the number of drugs and medication behavior [20, 21]. For example, Horne et al. reported that adherence is more influenced by the values that the patient places on their medicine than their characteristics [20]. Kamishima et al. reported that medication adherence of stroke patients decreased as the number of drugs increased [21] and that there are three characteristic in patients with poor adherence: 1) those who feel the number of medicines is too much, 2) those who have not received instruction by pharmacists and 3) those who feel anxiety taking medication for a long time [21]. When pharmacists introduce medication self-management for stroke patients, poor adherence is an important problem. Considering the above discussion, decreasing the number of drugs before hospitalization improves medicine self-management. This previous study supports the current results and increases the validity of the prediction formula.

In the current study, age was significantly lower in the self-management group than in the non-self-management group. Aging is associated with poor medication adherence caused by factors such as declining cognitive function and dysphagia [22, 23]. Accordingly, age is an important factor in considering introduction of medication self-management [22, 23].

In addition, the memory FIM item was associated with medicine self-management. This is similar to a previous study on achievement of medication self-management using the FIM [16–18]. The memory FIM item evaluates ability to memorize and reproduce linguistic and visual information in everyday life, a skill which is required to take medications correctly. In addition, it is also important to correctly understand pharmacist’s instructions, such as how and when to take a medicine. Accordingly, the FIM score is also an important factor in considering introduction of medication self-management.

This study has some limitations. The prediction formula was created for the patients in our hospital, but it was not subjected to external validation using data from other facilities. Therefore, there is a need to validate the formula if it is to be useful as a benchmark in other facilities. Furthermore, we did not examine the influence of the endpoint based on the rehabilitation program or that of patient education provided by pharmacists. Nonetheless, despite the limitations, the current prediction formula can be an effective tool to determine the likelihood of medication self-management in stroke patients at admission. Additionally, the formula may also help to prevent medication error.

## Conclusion

The number of drugs at admission greatly influenced achievement of medication self-management in stroke patients. In addition, the prediction formula developed herein may be useful to predict whether to introduce medication self-management for stroke patients.

## Abbreviations

FIM: Functional independence measure
LOF: Lack of fit
ROC: Area under the receiver operating characteristic
